# The Labor Productivity Consequences of Exposure to Particulate Matters: Evidence from a Chinese National Panel Survey

**DOI:** 10.3390/ijerph182312859

**Published:** 2021-12-06

**Authors:** Qi He, Xinde (James) Ji

**Affiliations:** 1 International Business School, Brandeis University, Waltham, MA 02453, USA; zjucamu@brandeis.edu; 2 Department of Economics, Brandeis University, Waltham, MA 02453, USA

**Keywords:** air pollution, particulate matter, labor productivity, CHNS

## Abstract

A growing body of literature has documented the negative impacts of air pollution on labor productivity, especially the effects of fine particulate matter. In this paper, we build on this literature by dissecting two channels of how particulate matter affects labor productivity: decreasing labor supply through damaging the physical functioning of the human body, and decreasing the marginal productivity of labor through damaging the cognitive functioning of the human brain. Using the household panel survey from the China Health and Nutrition Survey (CHNS) spanning 2000 to 2015 and combining that information with remotely sensed data on exposure to particulate matter (PM2.5), namely, the most harmful air pollution, we find a significantly negative effect of PM2.5 (instrumented by thermal inversion) on labor productivity. We also find that workers who are male, without a college degree, and are employed in outdoor occupations are mainly affected by PM2.5 through decreasing working hours, whereas college-educated workers employed in indoor occupations are mainly affected by PM2.5 through decreasing unit wages. We provide suggestive evidence that health impacts are behind our measured labor-productivity losses as we find significantly lower metrics in physical activity and increasing disease prevalence under higher exposure to PM2.5.

## 1. Introduction

Air pollution has become one of the greatest public health threats, with the most damaging air pollutant being fine particulate matter (PM2.5, i.e., any particles that have a diameter of 2.5 µm or less). Currently, 5.5 billion people around the world live in places that exceed the World Health Organization’s (WHO’s) safety guidelines for fine particulate matters. Research indicates that exposure to air pollution reduces the global average life expectancy by 1.8 years [1,2], and in the pollution-heavy China, by three years [3,4]. Studies have found exposure to fine particulate matter can result in physical health impairments, including respiratory and cardiovascular diseases such as impaired lung functioning, chronic obstructive pulmonary disease, asthma, or congestive heart failure [5,6,7,8]. At the same time, exposure to pollution also leads to impairment in brain functioning through systemic or brain oxidative stress and inflammation [9,10,11], which in turn leads to damaged cognitive functioning and dementia [12,13], and mental health problems [14,15].

A growing body of literature has also documented the socioeconomic impact of air pollution, especially in human capital and labor productivity. These studies showed productivity is negatively affected by exposure to air pollution in a variety of contexts, including test-takers in Israel [16], Brazil [17], and China [18], farmworkers in California [19], pear packers in Northern California [20], manufacturing firms in China [21,22], and call-center workers in China [23]. However, one point that is still unclear in the literature is the channels through which exposure to particulate matters affects productivity. Exposure to particulate matters may affect productivity from two distinct channels. First, it impairs the physical functioning of human bodies, including respiratory and cardiovascular diseases. This impairment will likely reduce the extensive margin of the labor supply, that is, limit the workers’ hours of work, requiring them to take longer breaks or drop out of the labor force temporarily or permanently. Second, exposure to air pollution also affects the cognitive function of human brains, which will likely reduce the intensive margin of labor productivity, that is, reductions in the quality of labor output per unit of working time. In a labor market equilibrium, this effect will eventually lead to decreases in workers’ unit wages as workers’ marginal products of labor decrease. Past studies on the productivity consequences of air pollution have primarily focused on the labor productivity consequences under a specific occupational context dominated by either physical functioning (e.g., [20,23]) or cognitive functioning [16,24]. As such, those studies have difficulty quantifying the effects on both margins and linking the estimated effects to their subjects’ underlying health conditions that drive the estimated labor market outcomes. 

In this paper, we quantify the labor market impacts from exposure to fine particulate matter, analyze heterogeneities of those impacts between the physical versus cognitive channels, and shed light on the underlying mechanisms in health conditions and subjective well-being metrics that drive the observed labor market outcomes. We use the household panel survey from the China Health and Nutrition Survey (CHNS), a nationally representative panel survey that tracks individuals’ health conditions and labor market outcomes for over a decade. (The respondents are randomly selected, and all of them use the same questionnaire) We match these individual-level data, covering 19,455 adults spanning 2000–2015, with data on exposure to particulate matter, weather conditions, and socioeconomic covariates. Following Graff Zivin and Neidell, we focus on three outcomes of interest: working months, which capture the longevity of a worker’s labor supply; annual working hours, which capture both the longevity and the intensity aspects of labor supply; and hourly wage, which captures a worker’s marginal productivity during his/her working time [25]. We choose annual working hours to reflect the overall working hours in the survey year. We also use working months to capture the seasonal nature of occupations such as farmers and fishers, who only work in certain months in a year. The last measure for productivity is individuals’ average hourly wage, which shows the marginal effect of productivity-ty in a different dimension from working hours.

There are three main empirical challenges in identifying the causal effect of air pollution on labor productivity. The primary empirical obstacle in estimation is reverse causality, such that higher individual productivity simultaneously leads to more industrial output and therefore more pollution, leading to an upward bias in the estimated effects [21]. The second empirical challenge is potential omitted-variable bias at the individual level, which may bias the estimated effect in both directions [26]. The last challenge is measurement error, such that air-pollution data might be manipulated [27]. To address these challenges, we use remote-sensing-based measures of particulate matter [28] and instrument that with the number of thermal inversion occurrence, a natural phenomenon that occurs when the temperature in the lower atmosphere is lower than that of the upper layer. In addition, we also add two-way fixed effects at the individual and year levels. Our identification relies on the assumption that thermal inversion is plausibly random, such that it is uncorrelated with most individual or regional characteristics that affect labor productivity or health outcomes.

Our empirical results suggest robust adverse effects of PM2.5 on labor productivity. On average, a 1 μg/m^3^ increase in PM2.5 concentration leads to 26.6 fewer working hours annually per person. It also decreases our subjects’ hourly wage by 0.34 yuan (USD 0.053) on average. We also find these results vary systematically by gender, education, and the nature of the employment. The impact of pollution exposure on working hours and months is more pronounced for men, rural residents, individuals without a college degree, and those engaging in low-skilled, outdoor occupations. This finding provides suggestive evidence in support of our hypothesis that the pollution impact through the physical-function channel mainly affects the supply of labor, that is, how long the worker can work in the short to medium term. On the other hand, the impact of pollution exposure on wages is more pronounced for men, people with a college degree, and individuals working in indoor occupations. This supports our hypothesis that the pollution impact through the cognitive-function channel mainly affects the marginal productivity of labor, that is, a worker’s effectiveness on the job. 

We further examine health mechanisms that potentially drive our estimated effects. We construct a version of the Quality of Well-Being (QWB) scale [29] using CHNS, comprehensively tracking an individual’s well-being in four distinct dimensions: mobility, physical activity, social activity, and disease symptoms. Consistent with prior literature [30,31], we find higher PM2.5 concentrations lead to lower metrics in physical activity, disease prevalence, and overall subjective well-being. We also find statistical evidence showing higher levels of exposure to particulate matter increases the probability of the residents having diabetes and asthma. 

This paper contributes to two distinct streams of literature. First, we contribute to the growing literature in public health and economics on the social impacts of air pollution. We quantify the causal effects of fine particulate matter on workers’ labor supply and wage outcomes in the context of the largest developing country, where over 90% of the citizens breathe air that is above the WHO safety guideline. Within a single labor-force sample (the CHNS), we seek to distinguish the effects of air pollution on physical versus cognitive functions. We shed light on two different channels, namely, the impairment of physical versus cognitive functions, through which air pollution can affect labor market outcomes. Past studies have primarily focused on identifying the impact of one such channel due to limitations in their respective study settings. 

Second, we contribute to the environmental justice literature on the differential impact of air pollution on the population from two angles. For one, we add to this literature by documenting the differential social impact of air pollution along the lines of existing social and economic inequalities. However, unlike most studies that focus on developed countries, a majority of the population we study are individuals who are relatively poor and are employed in agriculture or an otherwise blue-collar sector that work mostly outdoors. They are more likely to be directly exposed to air pollution and thus see impacts on their health and reductions in their capacity to work. We indeed find a substantial decrease in those workers’ labor supply and suggestive evidence that this decrease in working capacity is related to impaired physical activity and disease prevalence. The situation is comparable to the case in developed countries where the burden of exposure disproportionately falls on vulnerable groups within the society, but it is different in that the majority rather than the minority are experiencing the impact of reduced work capacity. We note here that more affluent, high-skilled, and college-educated workers are also affected by air pollution through the channel of decreasing wages, and the net wage impact differs across different divides. (We see low-skilled workers taking a heavier hit in total wage income than high-skilled workers. On the other hand, college-educated workers are hit heavier by air pollution in terms of total wage income, because their marginal productivity is more severely impacted. Table A6 presents those estimates). 

Our results also indicate a potential vicious trap of pollution exposure, where low-skilled workers are more likely to be exposed to air pollution and more likely to suffer from health consequences, and thus fall further down the social ladder in terms of income and welfare. This vicious trap has been documented in other settings, for example, the differential access among racial and income groups to air conditioning as an adaptation mechanism for climate-induced extreme heat [25,32,33], or migration and sorting into dirtier neighborhoods as a result of the income gap [34,35]. 

The remainder of the paper is organized as follows. Section 2 describes the data sources and summarizes the statistics. Section 3 introduces our empirical methods. Section 4 presents our main results. Section 5 provides discussions and concludes. 

## 2. Method 

The main goal of this paper is to estimate the causal effect of air pollution on labor productivity. Identification of the causal effect faces three empirical challenges. The primary empirical challenge is reverse causality. Higher economic output intensifies local air pollution, which in turn decreases labor productivity. Through this channel we would expect a negative relation between labor productivity and better ambient air quality at a local level [21]. Reversely, there is also empirical evidence suggesting regions with higher incomes are more likely to have stricter regulations, better enforcement, and a cleaner mix of industries with more advanced technologies in China [36,37]. Either way, typical linear regression (OLS) will result in biased estimates of pollution’s effect on labor productivity [21]. The second empirical challenge is potential omitted-variable bias, which may bias the main estimation in either direction. For instance, air pollution is typically positively correlated with confounders such as local economic conditions, which are in turn correlated with better healthcare infrastructure over time. In this case, the omitted factor causes an underestimation of pollution impact because better preventive and treatment measures are now available to residents, potentially leveling the negative effect of air pollution on productivity. The last challenge is measurement error in terms of pollution levels. References [27,38] provide cautionary tales on using monitoring-station data in China because those pieces of information may be subject to data manipulation. (Also see [39] for manipulation of monitoring-station data in the context of the U.S.) We address the last challenge by using remotely sensed pollution data provided by [28,40], which are largely free from manipulation. 

To address the first two main empirical challenges, we adopt the instrumental variable approach to identify the causal effect of air pollution on productivity. Specifically, we use thermal inversions to instrument for PM2.5 concentrations in our analysis, an instrument first proposed by [41]. Thermal inversion is a natural phenomenon whereby the temperature in the upper atmospheric layer is higher than the layers below. Airborne pollutants, including particulate matter, are thus trapped near the ground, leading to higher human exposure to air pollution. Because the formation of a thermal inversion is purely created by complex meteorological processes, it should be independent from other local socioeconomic factors that may affect pollution. This independence creates an exogenous variation in PM2.5 exposure, which we can use to instrument for air pollution. Studies have found a robust positive relationship between thermal inversion and local air-pollution exposure (e.g., [42], and have subsequently used thermal inversion to explore the effects of air pollution on various economic and social outcomes, including children’s health [43], migration [34,44], obesity [45], and mental health [14] (The relevance condition of the IV model is statistically examined using KP F-test [46] for weak instruments, which we present in the results section). Admittedly, we are unable to statistically test for the validity of the exclusion restrictions with a just-identified instrumental variable model. Thus, to provide an extra safeguard for the exogeneity of our instrument, we subsequently control for a variety of ground-level weather variables that may correlate with both air pollution and the formation of thermal inversion [47,48]. 

We propose the following panel-data regression framework, in which we regress different metrics for labor productivity on local levels of particulate matter. Our preferred estimation framework is thus given by the following instrumental variable approach, estimated via two-staged least squares (2SLS):

Y_ict = β_0 + β_1 [Pollution]_ct + f([Controls]_ict) + μ_i + v_t + ε_(ict)(1)

[Pollution]_ct = α_0 + α_1 [ThermalInv]_ct + g([Controls]_ict) + γ_i + σ_t + ε_ict(2)

where Y_ict denotes labor-productivity measures for individual i residing in county c in year t, including annual working hours, working months, and average hourly wage. [Pollution]_ct is the average annual PM2.5 in the year prior to the survey in county c, which matches up with the period of exposure for the reported working activities by our survey respondents. We instrument [Pollution]_ct using the total number of thermal inversion occurrences in the corresponding county and year, denoted by [ThermalInv]_ct in the first stage of the 2SLS model. As we discussed above, we include the same set of control variables in both stages of the estimation, include variables for 5 °C temperature bins, second-order polynomials in average snow thickness, vapor pressure, wind speed, sunshine duration, relative humidity, cumulative precipitation, rain duration, and snow duration. These local weather shocks have known effects on labor supply and productivity (e.g., [49,50]). Controls also include time-varying individual characteristics: retirement status and marital status, noting that any time-invariant individual characteristics will be absorbed by our individual fixed effect. Following Chen, Oliva, and Zhang (2018), we use individual fixed effects, μ_i, to control for any individual-specific characteristics such as gender, occupation, age, etc. We also include year fixed effects, v_t, to capture any time-specific shocks to productivity, such as business cycles, pollution-control policies, or national pandemics. ε_ict is the idiosyncratic error term, for which we employ two-way clustering at the county and year levels for all our models to control for autocorrelation in the measurements for the same county across different survey years [51].

## 3. Data

Our empirical analysis is facilitated by the CHNS, one of the most comprehensive longitudinal surveys that track individuals’ health, work, and nutritional status in multiple waves in China. The CHNS is jointly administered by the University of North Carolina at Chapel Hill and the Chinese Center for Disease Control and Prevention. We use six waves of the CHNS survey: years 2000, 2004, 2006, 2009, 2011, and 2015. The nationwide CHNS baseline survey in 2000 selected its samples using a multi-level clustered sampling method, interviewing 15,319 individuals selected from 225 communities located in 12 provinces. In 2015, the CHNS successfully followed up with 11,487 of the original individuals and added 3574 new individuals. (The new added individuals are randomly selected from the 225 communities.) The survey has been used in other studies on air pollution [45], dietary consumption [52], and environmental footprints (e.g., [53]. We then match individual responses in the CHNS with information on county-level averages for PM2.5 and climatic characteristics, using the six-digit community identifier provided by the CHNS (CNHS provides identifiers for province (two-digit code), city and prefecture (four-digit code), and county (six-digit code)).

The rest of the section documents our data-construction methods, starting from external datasets on air pollution, thermal inversion, and other climatic variables. We then document how we measure productivity and health outcomes from the CHNS. 

### 3.1. Air Pollution

We measure surface PM2.5 concentration using the regional annual PM2.5 reanalysis product[28,40]. (The dataset is available on the Atmospheric Composition Analysis Group website: http://fizz.phys.dal.ca/~atmos/martin/?page_id=140, accessed on 8 October 2020). The product measures PM2.5 using satellite-based aerosol optical depth (AOD) retrievals, which measures the amount of sunshine duration that is absorbed, reflected, and scattered by the particulates suspended in the air [54,55,56]. According to [40], AOD-retrieved surface PM2.5 concentration is highly accurate (R^2^ = 0.9–0.92). We obtain data with a spatial resolution of 0.01 × 0.01 degrees (approximately 1 km by 1 km).

### 3.2. Thermal Inversions

**Figure 1:**
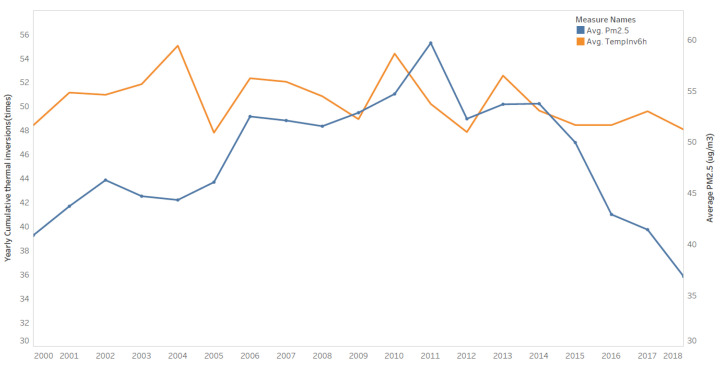
Average thermal inversion and PM2.5 over time. The orange line represents the average number of occurrences of 6 h thermal inversions over a year (scale on the left), and the blue line represents the annual average PM2.5 in μg/m^3^ (scale on the right).

We obtain the thermal-inversions data from NASA’s MERRA-2 project (Modern-Era Retrospective analysis for Research and Applications, Version 2, available online: https://doi.org/10.24381/cds.6c68c9bb, accessed on 3 March 2020). The data report six-hour air temperature for 42 atmospheric layers ranging from 110 m to 36,000 m, at a spatial resolution of 0.5 degree × 0.625 degree (around 50 km × 65 km). We follow Chen et al., (2018)’s method to calculate thermal inversion—comparing the temperature in the lowest layer (110 m) with the temperature in the second-lowest layer (320 m) for every six hours and count the occurrence of a thermal inversion over the entire year, that is, when the temperature in the second-lowest layer is higher than in the lowest layer, for each grid cell. Figure 1 plots the sample averages of thermal-inversion frequency and PM2.5 from 2000 to 2018. From a visual perspective, thermal inversion is positively correlated with the local level of PM2.5. 

### 3.3. Weather

Weather data are collected from AgERA5, a global atmospheric reanalysis dataset constructed by the European Center for Medium-Term Weather forecasting. (Available online: https://doi.org/10.24381/cds.6c68c9bb, accessed on 3 March 2020) Similar to the air-pollution data, AgERA5 has a spatial resolution of 0.01 × 0.01 degrees. We construct average annual climate data, including temperature bins, total precipitation, total snow thickness, and cloud cover, by averaging over all grid points in each county [57,58]. Following past literature [14,32], we count the number of days in the year within each 5 °C interval using daily average temperature, in order to control for the potentially non-linear impacts of temperature on productivity. 

### 3.4. Labor Productivity

We measure survey respondents’ labor productivity at two margins: (1) the amount of time they work and (2) their unit wage. We expect air pollution to affect both margins. On the time margin, severe air pollution could reduce a worker’s ability to work for longer hours in a prolonged period of time by negatively affecting the worker’s health. We expect the impact to be more pronounced for outdoor, labor-intensive occupations. We also explore the health-pollution linkage in the mechanism section. On the wage margin, air pollution could also affect a worker’s ability to perform both labor-intensive and cognition-intensive tasks, thus reducing their marginal product of labor. 

We construct three variables as our outcomes of interest: (1) annual working hours, that is, the aggregate amount of time a worker has worked in the past year (we calculate the annual working hours by multiplying the responses from three survey questions: the average number of days per week worked last year, the average number of months worked last year, and the average number of hours per day worked last year.) (2) average working months, i.e., the number of months a respondent worked in the past year, which help us dissect the potential impact of seasonal workers; and (3) the average hourly wage, inflation-adjusted to the year 1999. CHNS documents monthly salary for each survey respondent, and we calculate hourly wage by: hourly wage = monthly wage/working hours per day × working days per week × 4 weeks per month). We also conduct robustness checks by using monthly salary as the outcome variable, noting that monthly salary may also pick up some of the labor supply effects. The results are very similar. We obtained the data for the GDP deflator in China from the world bank, (https://databank.worldbank.org/reports.aspx?source=world-development-indicators, accessed on 23 December 2020). Then, we applied the obtained deflation rate to convert the wages in each year to 1999 yuan. The first two variables measure potential changes in labor supply [21], and the third measures potential changes in labor productivity. The sample sizes for these variables are different due to some respondents did not report annual hours worked or their hourly wage.

### 3.5. Human Health

We construct measures on human health from the CHNS, available from 2000 to 2011 (the 2015 wave is not covered in the CHNS). The CHNS includes 215 questions about individuals’ health status, and our goal is to reconcile these individual measures into a universal metric. We adopt the framework of QWB, a metric widely used and extensively validated in the medical literature as a comprehensive measure of health-related quality of life, and robust to multiple underlying diseases, such as lung disease, tumor/cancer, and asthma [29,59,60,61,62]. The QWB scale is shorter, cheaper, and more sensitive to changes in disease symptoms than other measures of human health such as the Sickness Impact Profile, (SIP), the Health Utilities Index Mark 3 (HUI3), and the Assessment of Quality of Life (AQOL).

We follow Kaplan, Atkins, and Timms’s (1984) and Zhao and Hou’s (2005) method in constructing the QWB scale, which contains four indexes: mobility scale (MOB), physical activity scale (PAC), social activity scale (SAC), and symptom/problem complex (CPX). We calculate the four indices based on our subjects’ answers and weigh their answers based on guidance from [63]. We aggregate using W = 1 + CPX + MOB + PAC + SAC (See questions and weights provided in Appendix A Table A1). The QWB scale then provides a numerical point-time expression of well-being, ranging from 0 when a respondent is deceased to 1 when a respondent is asymptomatic and fully functional. In addition to the QWB index, we also construct disease-specific indicator variables for high blood pressure, diabetes, cancer, and asthma. 

### 3.6. Summary Statistics

**Figure 2:**
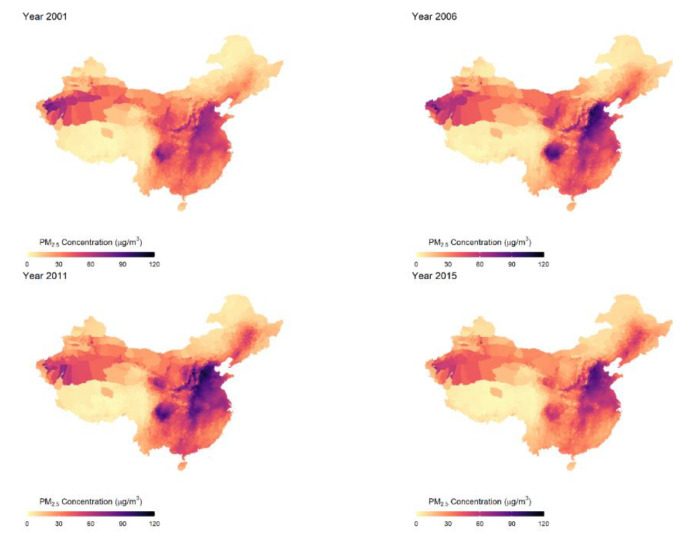
Average Annual PM2.5 Concentration by county. The (**top-left**) panel shows the annual average PM2.5 concentration (in μg/m^3^) in year 2001; the (**top-right**) panel maps the year 2006; the (**bottom-left**) panel maps the year 2011; the (**bottom-right**) panel maps the year 2015. Source: Authors’ calculation.

Table 1 presents our summary statistics, and Figure 2 maps the spatial variation in PM2.5 concentrations at the county level (in μg/m^3^) for the years 2001, 2006, 2011, and 2015. PM2.5 concentration increased in 2006 and 2011, followed by a decline in 2015 due to the anti-pollution campaign starting in the 2010s [64]. Still, the average level of PM2.5 exposure in our sample is 51.3 μg/m^3^, five times more than the WHO’s annual safety guideline [1]. In the meantime, the spatial distribution of PM2.5 exposure is highly heterogeneous: the Northeastern part of China, including Beijing, Tianjin, Hebei, Henan, and Shanxi, experienced much higher exposure to fine particulate matter than the rest of the country, partly due to the centralized winter-heating program that ran on coal [3,4]. The highest PM2.5 exposure appears in Henan province in 2011, reaching an astonishing 107 μg/m^3^, more than 10 times above the WHO guideline. 

## 4. Results

### 4.1. Effect of Thermal Inversions on PM2.5 Concentrations

We start by demonstrating the relevance of our instruments, that is, how thermal inversions correlate with PM2.5 concentrations in the first-stage equation of Equation (2). Table 2 reports the estimated effect of thermal inversions on PM2.5 concentrations. All three models’ estimations include individual and year fixed effects and all other control variables except for PM2.5, identical to our main specification. 

We find a significant positive relationship between the prevalence of thermal inversions and PM2.5 concentrations, suggesting that thermal inversion increases local exposure to particulate matter. A one-standard-deviation increase in the occurrence of thermal inversion increases average PM2.5 concentrations by 0.66 standard deviation, or 13.0 μg/m^3^, which is statistically significant at the 1% level. We report Kleibergen-Paap’s F-statistic [65] in Table 2. All the KP values in columns (1)–(3) are larger than the critical value of 16.38 for the Stock-Yogo weak identification test [46]. This suggests thermal inversion satisfies the relevance condition as an instrument for PM2.5.

### 4.2. Instrumental Variable Estimates of the Effect of PM2.5 Concentrations on Productivity

We now turn to the main result of the paper. Table 3 reports the estimates of the impact of air pollution on various measures of labor productivity. The dependent variables are annual working hours in columns (1) and (2), monthly working hours in columns (3) and (4), and average hourly wage in columns (5) and (6). Columns (2), (4), and (6) report the instrumental variable estimates using 2SLS. We also include columns (1), (3), and (5) to report the fixed effects estimates when air pollution is not instrumented (OLS). All columns include two-way fixed effects by individual and year, and we report robust standard errors two-way clustered at the county-year level.

In contrast with the 2SLS estimates, the OLS coefficient estimates are positive and statistically insignificant. They are also significantly different from the nested 2SLS estimates, suggesting that without instrumenting for air pollution, the OLS estimates will be biased upward. Thus, we interpret our results below using the 2SLS estimates. 

We find a statistically significant negative effect of PM2.5 concentrations on labor supply. In column (2), our estimates suggest a one μg/m^3^ increase in PM2.5 concentration leads to a decrease of 26.6 working hours, which is significant at the 1% level. A one-standard-deviation increase in PM2.5 concentration decreases annual working hours by 515.0 h, which is about half of a standard deviation decrease in annual working hours. We also find PM2.5 concentrations have a negative effect on working months. Column (4) shows a one μg/m^3^ increase in PM2.5 concentration leads to a decrease of 0.082 months worked in the past year, which is significant at the 5% level. In other words, a one-standard-deviation increase in PM2.5 concentrations decreases working months by 0.48 standard deviations. Combining those two outcomes together, we find that about half of the labor-supply effects come from decreases in the duration of employment (−0.082 months, or −12.8 h per year), and the rest of the effect comes from decreases in the intensity of employment, that is, within a month (−1.16 h per month, or −13.8 h per year).

We also find exposure to air pollution reduces workers’ marginal productivity, measured by a decrease in wages. Column (6) suggests a negative relationship between hourly wage and PM2.5 concentrations: a one μg/m^3^ increase in PM2.5 concentrations decreases the hourly wage by 0.34 yuan (USD 0.053), which is statistically significant at the 5% level. The point estimates indicate that a one-standard-deviation increase in PM2.5 concentrations leads to a decrease of 6.58 RMB, or a 0.49-standard-deviation decrease, in hourly wages. We have tested the impacts of air pollution on labor productivity only for the 9175 respondents who have reported all three measures, the results are shown in Table A3, it stays very consistent with our main results. Selected control variables are reported in Table A4. To be specific, we expect retirement to be negatively affecting labor supply, but has no impact on wages; extreme weather such as extreme hot and cold days will lead to less productivity.

### 4.3. Heterogeneity

We now turn to analyze the heterogeneous effects of air pollution on labor productivity by breaking down our sample by demographics, education, and type of employment. All models are run with 2SLS with thermal inversion as the instrumental variable, identical to our main model. 

We first report results broken down by demographics and education, reported in Table 4. We start with the differential impact of air pollution by gender. Male respondents account for 58% of our sample, and they generally worked longer hours and more months and received a higher hourly wage. Columns (1) and (2) of Table 4 show the estimated impact of air pollution by gender. We find pollution disproportionately affects male workers, reducing their annual working hours by 32 h, working months by 0.12 months, and hourly wages by 0.37 RMB (USD 0.06). All three estimated outcome variables are statistically significant. On the contrary, female workers are virtually not affected by air pollution: all three outcome variables are statistically insignificant. These results are consistent with previous findings on the differential impact of air pollution by gender, such that both infant and adult males are more sensitive to the exposure of air pollution [12,66,67].

We then explore the heterogeneity by educational level in columns (3) and (4). We divide the sample into two groups: 19% of our respondents have attended college, and the remaining 81% have not. For college graduates, a one μg/m^3^ increase in PM2.5 decreases annual working hours by 43 h and working months by 0.033 months, though both effects are statistically insignificant. On the other hand, we find a statistically significant negative impact of air pollution on college-educated workers’ hourly wages. In comparison, air pollution has a statistically significant negative effect on non-college-educated workers’ working time, but we find no statistical evidence to show air pollution can affect their hourly wages. One reason that could explain this difference is the nature of occupation across educational groups [45]. Relative to non-college-educated workers, college-educated workers are more likely to work in sectors that require cognitive functioning rather than physical functioning and receive much higher hourly wages (9.45 RMB) than non-college-educated workers (6.23 RMB). Although evidence has suggested that air pollution affects both the physical and the cognitive functioning of human beings, we expect non-college-educated workers to be affected mainly through physical-functioning impairments, which reduces their working hours. On the other hand, college-educated workers are mainly affected by cognitive-functioning impairments, reflected by a wage decrease. The estimation results match our expectations well, and this hypothesis is further corroborated when we break down the impact of pollution by occupation. As a robustness check, we have divided the data into five groups based on the individuals’ highest level of education attained. The estimation results are shown in Table A5. It splits the individuals by graduation from primary school, middle school, high school, technical or vocational school, or college and above. The results are very consistent with our main estimation and showing that air pollution is more likely to have negative impacts on less educated people’s working time and more educated people’s hourly wage.

The last two columns (columns (5) and (6)) focus on the heterogeneity of urban and rural residencies. Urban individuals account for 28.5% of our sample. Our estimation results suggest that PM2.5 concentrations have a negative impact on all three measures for both rural and urban residencies. However, the estimates are only significant for rural residents’ working time. The underlying mechanism is likely similar to the college-educated and non-college-educated divide: on average, rural residents conduct more physical work, whereas urban residents are more likely to engage in white-collar, cognition-heavy jobs.

We continue our breakdown in Table 5, which reports estimates for the subsamples divided by the nature of employment. Columns (1) and (2) in Table 5 report the breakdown between high-skilled occupations, including senior professional/technical worker, army officer, police officer, foreman, and group leader in column (1), versus the rest of the occupations, presented in column (2). The correlation between skill and college education in our sample is modest, which explains why there are subtle differences between the subsample analysis along the skills vs. educational divide. Many of our survey respondents are employed by the public sector, including the government, public institutions (universities, hospitals, etc.), and state-owned enterprises (SOEs). Part of the variations of the high skills vs. low skill occupations we adopted here came from our respondents’ respective rank in the public sector. Many high skilled workers rose through the ranks without obtaining a college degree, for example, army and police officers, skilled laborers, village leader, etc. Our results suggest higher PM2.5 concentrations have no significant effect on our three measures of high-skilled workers’ productivity. On the other hand, we find significant decreases in working time as a result of increasing air-pollution exposure of workers in low-skilled occupations. 

In columns (3) and (4) of Table 5, we present results broken down by indoor versus outdoor occupations. Outdoor-only occupations include fisher, farmer, hunter, soldier, police officer, and driver, presented in column (4). Indoor occupations include the remaining occupations, presented in column (3). These results are consistent with our previous story: indoor occupations are mainly affected by air pollution through decreases in wages, whereas outdoor occupations are mainly affected by reductions in working hours. This observation lines up with our expectation: studies have repeatedly shown indoor occupations are affected more by air pollution through impairment in cognitive functions [23], whereas outdoor workers are more affected by physical activities that restrict their working time. 

### 4.4. Robustness Checks

In this subsection, we document efforts to check the robustness of our results. Alternative model specifications are presented in Table 6, where column (1) presents our baseline specification. We first test the robustness of our instrumental variable, thermal inversions. In columns (2) and (3), we replace the number of thermal inversions aggregated by a 12 h window and a 24 h window instead of the 6 h window used in our baseline model. The results are similar to our baseline model that air pollution has a negative impact on both individuals’ working time and hourly wage.

We also report temporal falsification tests in columns (4) and (5). In our main model, the exposure window is aggregated at the year of the survey, that is, one year prior to the reported wave year. In columns (4) and (5), we use pollution-exposure windows 12 months before and after the survey year as alternative exposure windows. If results from the alternative exposure windows are statistically significant, that could indicate that our modeling approach is potentially mis-specified. Coefficient estimates from both the one-year-lag and the one-year-lead model for all three outcomes are statistically insignificant, which alleviates concerns for misspecification and lends support to the validity of our empirical results. The insignificant coefficients in column (4) also suggest the impact of air pollution on individuals’ working time and hourly wage tend to be short-lived. In other words, exposure to air pollution in the prior year does not have significant impacts on individuals’ working time or hourly wage in the current year. 

### 4.5. Mechanisms

In this subsection, we shed light on the potential mechanisms that cause air pollution to affect labor productivity. Specifically, we posit that pollution negatively affects human health, which then reduces workers’ capacity to perform both physical and cognitive tasks. We proxy health status using the QWB scale, which consists of four components: MOB, PAC, SAC, and CPX. We also examine whether exposure to air pollution has any impact on the occurrence of high blood pressure, diabetes, and asthma. We match all the health measurements to the corresponding survey year. 

Table 7 reports our results. Columns (1) to (5) report the effect of PM2.5 concentration on health indexes. We find a one μg/m^3^ increase in PM2.5 concentration will decrease the overall QWB index by 0.019. Decomposing QWB into subcategories, a one μg/m^3^ increase in PM2.5 concentration decreases PAC by 0.003 and CPX by 0.002. We do not find statistically significant impacts on MOB or SAC. Overall, our results provide suggestive evidence that higher levels of air pollution lead to impairments in physical activity, increases in disease symptoms, and worse overall health conditions. This is logically consistent with our finding that air pollution reduces working duration and intensity, especially for outdoor occupations and occupations that require physical functioning. 

Columns (6) to (8) report the effect of PM2.5 concentrations on the occurrence of physical illnesses, including high blood pressure, diabetes, and asthma. All three dependent variables are equal to 1 if the individual has been diagnosed with the disease in the year of the survey, and 0 otherwise. We find no statistical evidence to show PM2.5 concentrations can affect the probability of high blood pressure. However, we find a one μg/m^3^ increase in local PM2.5 concentration increases the probability of diabetes and asthma by 0.003 and 0.001, respectively. Both effects are significant at the 5% level. These findings are consistent with previous studies in physiological and medical science that more severe air pollution leads to more diabetes and asthma [5,6]. 

## 5. Discussion and Conclusions

Using the CHNS dataset that has tracked 22,000 individuals in China for over a decade, we estimate the effect of PM2.5 concentration on labor productivity. By leveraging thermal inversion as an instrumental variable, we address the main empirical challenges in the estimation procedure, including reverse causality and potential omitted-variable bias. Our results indicate a statistically significant negative relationship between PM2.5 exposure and labor productivity. Higher air pollution levels lead to a significant decrease in both the physical supply of labor in working hours and the productivity in average wages. We further benchmark our estimates with previous studies that estimate the effect of air pollution on labor productivity. For example, Chang et al. use pear packers in the U.S. and report a 10 μg/m^−3^ decrease in PM2.5 concentration increases monthly earnings by 15% of a standard deviation [20]. In [23], a 10-unit increase in API decreases the number of calls by 0.35%. Additionally, reference [22] find a 1% decrease in workers’ daily output after a 10 μg/m^−3^ increase in PM2.5 over a 25-day window. Our estimated effects are larger than those estimates mentioned above but are within the same order of magnitude, our larger estimates may result from the fact that while these studies measure the effect of short-run, that is, daily to monthly, exposures, whereas we capture the medium-run impact of annual average exposure here.

At the same time, we find significant heterogeneities among different population groups with respect to pollution impact, especially between outdoor, physical-intensive occupations versus indoor, cognitive-intensive occupations. We find non-college-educated subjects in physical-activity-heavy occupations are mainly affected through the channel of reduced working hours and not by reductions in average wages. This observation is consistent with the findings in Chang et al., in which the reduction in productivity for call-center workers is mainly attributable to the extensive margin on the increasing amount of time spent on breaks rather than the intensive margin on the average duration of calls [23]. On the other hand, highly educated subjects in cognitive-heavy occupations are mainly affected by air pollution through wage decreases rather than a reduction in working hours, which is consistent with the literature showing air pollution also impairs cognitive functioning [13,17,24]. 

Our empirical approach can be readily applied to other regions around the world given the availability of health and economic outcomes with proper geographical references, especially in places where environmental monitoring stations are sparse and pollution levels are hard to pin down. We leverage gridded datasets on air pollution and weather conditions, the former remotely sensed, which provides relatively accurate measures of environmental conditions while also offering safeguards against tampering with environmental statistics, a phenomenon existing in both developed and developing countries [27,39,68]. 

One limitation of this study is that because the CHNS is only available at the annual level, we identify our effects using yearly variations in air pollution and labor productivity. As such, we are not able to distinguish between the effect of acute exposure to air pollution that other papers have documented [7,69]. Nevertheless, we believe documenting the effect of sustained air-pollution exposure and its medium and long-run impacts is still meaningful, especially for developing countries, such as China and India, where air-pollution levels far exceed the WHO guidelines for a sustained period of time each year [69]. 

Particulate matter is already one of the greatest threats to human health. At the same time, it puts a significant burden on individuals in terms of human capital, labor productivity, and quality of life. Therefore, measures that aim to improve air quality, such as transitioning to clean energy or restricting agricultural residual burning, have the potential to provide significant health, economic, and social benefits to individuals, firms, and society around the world. Apart from public actions, our study also provides a rationale for individuals to enact defensive actions that guard against air-pollution exposure through facemasks [70], air purifiers [71], or simply staying home [72]. However, these defensive actions might not be possible and/or affordable to all groups facing air-pollution exposure, and the burden falls disproportionately on low-skilled workers engaged in outdoor, physical-heavy occupations and who lack the will or resources to engage in defensive measures. With the growing trend of working from home for skilled workers, we expect the gap in pollution exposure to widen further along existing socioeconomic inequalities.

## Figures and Tables

**Table 1:** Summary statistics.

Variable	Description	N	Min	Max	Mean	SD
County	County	225				
Year	2000, 2004, 2006, 2009, 2011, 2015	6				
Productivity Variables						
AWH	Annual working hours	25,452	0	8064	1423.37	1070.96
Month	Working months	26,068	0	12	9.76	3.24
Wage	Average hourly wage (1999 yuan)	12,401	0.13	427.17	6.98	13.26
Health Variables						
QWB	Quality of well-being	14,244	0.32	1	0.68	0.20
MOD	Mobility scales	14,579	−0.09	0	−0.05	0.04
PAC	Physical activity scales	14,470	−0.77	0	−0.03	0.03
SAC	Social activity scales	46,495	−0.11	0	−0.01	0.03
CPX	Symptom/problem complexes	64,564	−0.41	0	−0.06	0.12
HB	High blood pressure patient = 1	64,564	0	1	0.08	0.28
Diabetes	Diabetes patient = 1	64,564	0	1	0.02	0.14
Cancer	Cancer patient = 1	13,032	0	1	0.01	0.11
Asthma	Asthma patient = 1	23,752	0	1	0.01	0.11
Air pollution						
PM_2.5_	Fine particulate matter concentration (μg/m^3^)	64,564	6.27	110.60	49.77	18.54
Climate Variables						
Inversions6 h	Times in 12 months (over 6 h)	64,564	0	143.75	48.64	39.40
Inversions12 h	Times in 12 months (over 12 h)	64,564	0	213	81.68	65.87
Inversions24 h	Times in 12 months (over 24 h)	64,564	0	282	111.89	87.25
Temperature	Temperature at the surface (°C)	64,564	12.62	37.74	12.62	11.38
Rain	Precipitation at the surface (mm/hour)	64,564	36.37	264.34	113.89	58.49
Wind speed	Windspeed 10 m	64,564	1.52	5.26	2.90	0.58
Snow	Snow thickness (mm)	64,564	0	2346.54	129.18	364.17
Individual characteristics						
Marital status	Married = 1; Otherwise = 0	24,817	0	1	0.96	0.19
Retirement	Retired = 1; Otherwise = 0	29,350	0	1	0.14	0.36

Notes: Unit of observation is individual year. The survey covered 15,319 adult individuals (age ≥ 18) from 225 counties across 12 provinces during 2000–2015 in China. QWB-scale indexes are calculated by authors. The variables measure the individuals’ health condition one year prior to the survey year. Thermal inversion is determined within each 6 h period, 12 h period, 24 h period, and then aggregated to 12 months.

**Table 2:** First-sage estimation: effect of thermal inversions on PM2.5 concentrations.

		PM_2.5_(μg/m^3^)	
	(1) Annual Working Hours	(2) Working Months	(3) Average Hourly Wage
Thermal inversions	0.268 *** (0.010)	0.270 *** (0.009)	0.247 *** (0.057)
*R*-squared	0.70	0.70	0.54
Individual FE	Yes	Yes	Yes
Year FE	Yes	Yes	Yes
Individual control	Yes	Yes	Yes
Weather controls	Yes	Yes	Yes
KP *F*-statistic	31.96	34.02	35.03
Observations	18,346	18,711	9376

Notes: The dependent variable is annual local county PM2.5 concentrations in the last year. We exclude individuals whose hourly wage (1999 Yuan) is above 430 (the top 0.3%) or below 0.128 RMB (the bottom 0.3%) in column (3) to avoid outlier bias. Thermal inversions are aggregated from every 6 h to 12 months for the last year. Weather controls include 5 °C temperature bins, second-order polynomials in average snow thickness, vapor pressure, windspeed, sunshine duration, relative humidity, cumulative precipitation rain duration, and precipitation solid duration for the last year. Individual controls include variables indicating the individual’s marital status, retired or not, etc. Standard errors are listed in parentheses and clustered by both county and year (two-way clustering). *** *p* < 0.01. The numbers of observations in columns (1)–(3) are different because of missing dependent variables, especially hourly wage.

**Table 3:** Second-stage estimation: effect of PM2.5 concentrations on productivity.

	Annual Working Hours		Working Months		Average Hourly Wage	
	(1) OLS	(2) 2SLS	(3) OLS	(4) 2SLS	(5) OLS	(6) 2SLS
PM_2.5_ (μg/m^3^)	5.656 (3.851)	−26.60 *** (10.09)	0.020 (0.013)	−0.082 ** (0.033)	−0.009 (0.053)	−0.34 ** (0.17)
KP F-statistic		63.92		68.04		40.97
Mean of Dep	1653	1653	9.760	9.760	6.98	6.98
S.D. of Dep	977.8	977.8	3.242	3.242	13.26	13.26
Individual FE	Yes	Yes	Yes	Yes	Yes	Yes
Year FE	Yes	Yes	Yes	Yes	Yes	Yes
Weather controls	Yes	Yes	Yes	Yes	Yes	Yes
Individual controls	Yes	Yes	Yes	Yes	Yes	Yes
Observations	18,346	18,346	18,711	18,711	9376	9376

Notes: The dependent variables are annual working hours in the last year in columns (1) and (2), working months last year in columns (3) and (4), and average hourly wage last year in columns (5) and (6). We exclude individuals whose hourly wage (1999 Yuan) is above 430 (the top 0.3%) or below 0.128 RMB (the bottom 0.3%) in columns (5) and (6) to avoid outlier bias. Columns (1), (3) and (5) report the OLS estimates in which air pollution is not instrumented. Columns (2), (4) and (6) report 2SLS estimates, in which we use the number of thermal inversions to instrument for PM2.5. Weather controls include 5 °C temperature bins, second-order polynomials in average snow thickness, vapor pressure, windspeed, sunshine duration, and relative humidity, cumulative precipitation rain duration, and precipitation solid duration for the last year. Individual controls include variables indicating the individual’s marital status, retired or not, etc. Standard errors are listed in parentheses and clustered by both county and year (two-way clustering). *** *p* < 0.01, ** *p* < 0.05.

**Table 4:** Effect of air pollution on productivity: by gender, educational attainment, and residence.

	Gender		Education		Residence	
	(1) Male	(2) Female	(3) <College	(4) ≥College	(5) Urban	(6) Rural
**Annual working hours**						
PM_2.5_ (μg/m^3^)	−32.03 *** (11.42)	−15.92 (12.79)	−25.54 ** (10.88)	−43.26 (25.20)	10.63 (15.34)	−24.71 * (12.76)
Mean of Dep	1712.2	1589.54	1634.37	1750.68	2002.30	1505.89
S.D. of Dep	963.2	989.08	1005.86	810.9	811.95	1004.21
KP F-statistic	61.76	56.34	61.31	62.92	24.21	64.93
Observations	10,144	8202	16,731	1615	5124	13,222
**Working months**						
PM_2.5_ (μg/m^3^)	−0.118 *** (0.037)	−0.029 (0.043)	−0.091 ** (0.037)	−0.033 (0.048)	−0.010 (0.037)	−0.078 * (0.044)
Mean of Dep	9.853	9.658	9.613	10.533	10.959	9.257
S.D. of Dep	3.179	3.308	3.291	2.853	2.485	3.388
KP F-statistic	59.44	58.73	63.45	43.38	23.48	66.71
Observations	10,341	8370	17,088	1623	5210	13,501
**Hourly wage**						
PM_2.5_ (μg/m^3^)	−0.37 ** (0.018)	−0.17 (0.25)	−0.16 (0.18)	−1.03 ** (0.42)	−0.15 (0.19)	−0.63 (0.44)
Mean of Dep	7.73	6.01	6.23	9.45	7.14	6.86
S.D. of Dep	13.72	13.10	12.43	15.41	12.29	14.02
KP F-statistic	41.51	30.17	39.93	24.60	24.93	25.43
Observations	5144	4232	7068	2308	4296	5080

Notes: The dependent variables are annual working hours in the last year in Section 1, working months in the last year in Section 2, and average hourly wage in the last year in Section 3. To avoid outlier bias, we exclude individuals who earn the top 0.3% and the bottom 0.3% in Section 3 (hourly wage). Regression models are estimated separately for each subsample. All the regressions report 2SLS estimates with controls. Weather controls include 5 °C temperature bins, second-order polynomials in average snow thickness, vapor pressure, windspeed, sunshine duration, relative humidity, cumulative precipitation rain duration, and precipitation solid duration for the last year. Individual controls include variables indicating the individual’s marital status, retired or not, etc. Standard errors are listed in parentheses and clustered by both county and year (two-way clustering). *** *p* < 0.01, ** *p* < 0.05, * *p* < 0.1.

**Table 5:** Effect of air pollution on productivity: by nature of occupation.

	Skills		Workplace	
	(1) High Skilled	(2) Low Skilled	(3) Indoor	(4) Outdoor
**Annual working hours**				
PM_2.5_ (μg/m^3^)	−3.642 (12.63)	−31.86 ** (12.62)	2.499 (10.08)	−27.53 * (13.39)
Mean of Dep	2005.48	1617.236	1970.03	1364.28
S.D. of Dep	565.94	1003.591	753.70	1065.47
KP F-statistic	38.71	58.93	36.57	65.76
Observations	1717	16,629	7439	10,907
**Working months**				
PM_2.5_ (μg/m^3^)	−0.002 (0.022)	−0.102 ** (0.038)	0.012 (0.023)	−0.087 * (0.050)
Mean of Dep	11.712	9.563	10.898	8.735
S.D. of Dep	1.384	3.310	2.422	3.530
KP F-statistic	29.06	61.43	37.26	47.51
Observations	1732	16,979	7509	11,202
**Hourly wage**				
PM_2.5_ (μg/m^3^)	−0.23 (0.19)	−0.49 (0.31)	−0.51 *** (0.20)	0.09 (0.37)
Mean of Dep	8.66	6.62	7.03	6.81
S.D. of Dep	13.48	13.19	11.99	16.90
KP F-statistic	25.46	35.52	38.54	29.04
Observations	1874	7502	7017	2359

Notes: The dependent variables are annual working hours in the last year in Section 1, working months the last year in Section 2, and average hourly wage in the last year in Section 3. To avoid outlier bias, we exclude individuals who earn the top 0.3% and the bottom 0.3% in Section 3 (hourly wage). Regression models are estimated separately for each subsample. Column (1) estimates the high-skilled laborers, including senior professionals/technical workers, army officers, police officers, forepersons, and group leaders. Column (2) uses the data for the remaining jobs. In column (4), we present the regression results for respondents whose workplace is outdoors only, including fishermen, farmers, hunters, soldiers, police officers, and drivers. We focus on the remaining respondents whose workplace is indoors only in column (3). All the regressions report 2SLS estimates with controls. Weather controls include 5 °C temperature bins, second-order polynomials in average snow thickness, vapor pressure, windspeed, sunshine duration, relative humidity, cumulative precipitation rain duration, and precipitation solid duration for the last year. Individual controls include variables indicating the individual’s marital status, retired or not, etc. Standard errors are listed in parentheses and clustered by both county and year (two-way clustering). *** *p* < 0.01, ** *p* < 0.05, * *p* < 0.1.

**Table 6:** Robustness checks.

	(1) Baseline	(2) TI in 12 h	(3) TI in 24 h	(4) Lag 12 Months PM_2.5_	(5) Lead 12 Months PM_2.5_
**Annual working hours**					
PM_2.5_ (μg/m^3^)	−26.60 *** (10.09)	−31.12 ** (12.53)	−26.22 ** (10.33)	4.354 (18.39)	−38.54 (40.09)
KP F-statistic	63.92	66.21	68.90	72.83	64.05
Observations	18,346	18,346	18,346	18,346	18,346
**Working months**					
PM_2.5_ (μg/m^3^)	−0.082 ** (0.033)	−0.107 ** (0.042)	−0.074 ** (0.037)	0.036 (0.070)	−0.177 (0.169)
KP F-statistic	68.04	68.08	70.51	69.50	73.07
Observations	18,711	18,711	18,711	18,711	18,711
**Hourly wage**					
PM_2.5_ (μg/m^3^)	−0.34 ** (0.17)	−0.36 * (0.21)	−0.33 * (0.17)	−0.24 (0.41)	0.16 (0.45)
KP F-statistic	20.16	27.98	32.18	23.73	28.71
Observations	9376	9376	9376	9376	9376

Notes: The dependent variables are annual working hours in the last year in Section 1, working months in the last year in Section 2, and average hourly wage in the last year in Section 3. To avoid outlier bias, we exclude individuals who earn the top 0.3% and the bottom 0.3% in Section 3 (hourly wage). Regression models are estimated separately for each subsample. All the regressions report 2SLS estimates with controls. Weather controls include 5 °C temperature bins, second-order polynomials in average snow thickness, vapor pressure, windspeed, sunshine duration, relative humidity, cumulative precipitation rain duration, and precipitation solid duration for the last year. Individual controls include variables indicating the individual’s marital status, retired or not, etc. Standard errors are listed in parentheses and clustered by both county and year (two-way clustering). Column (1) is the baseline model. Columns (2) and (3) replaces TI counts with 12 and 24 h rather than 6 h, which is used in the baseline model, respectively. Column (4) uses a lag of 12 months of PM2.5 as the exposure window, and column (5) uses a lead of 12 months of PM2.5 as the exposure window. *** *p* < 0.01, ** *p* < 0.05, * *p* < 0.1.

**Table 7:** Subjective well-being as mechanisms.

	QWB Indices					Disease Prevalence		
	(1) MOB	(2) PAC	(3) SAC	(4) CPX	(5) QWB	(6) Suffer High Blood Pressure = 1	(7) Suffer Diabetes = 1	(8) Suffer Asthma = 1
PM_2.5_ (μg/m^3^)	−0.0003 (0.0007)	−0.0031 *** (0.0012)	−0.0003 (0.0002)	−0.0024 ** (0.0011)	−0.0190 ** (0.009)	0.0077 (0.0091)	0.0032 ** (0.0015)	0.0010 ** (0.0005)
Individual FE	Yes	Yes	Yes	Yes	Yes	Yes	Yes	Yes
Year FE	Yes	Yes	Yes	Yes	Yes	Yes	Yes	Yes
Weather controls	Yes	Yes	Yes	Yes	Yes	Yes	Yes	Yes
Individual controls	Yes	Yes	Yes	Yes	Yes	Yes	Yes	Yes
Mean of Dep	−0.055	−0.029	−0.009	−0.073	0.637	0.105	0.023	0.012
S.D. of Dep	0.038	0.033	0.027	0.132	0.180	0.306	0.149	0.111
KP F-statistic	16.57	16.55	67.06	67.61	22.33	291.82	195.16	128.67
Observations	6590	6585	21,211	51,123	6585	45,493	36,577	13,469

Notes: Dependent variables are five QWB indexes in columns (1)–(5). A dummy variable equals 1 if the individual has been diagnosed with the corresponding diseases in columns (6)–(8) and equals 0 otherwise. Therefore, we adopt a probit model to estimate the effects of PM2.5 on the diseases. All the regressions report 2SLS estimates with controls. Weather controls include 5 °C temperature bins, second-order polynomials in average snow thickness, vapor pressure, windspeed, sunshine duration, relative humidity, cumulative precipitation rain duration, and precipitation solid duration for the last year. Individual controls include variables indicating the individual’s marital status, retired or not, etc. Standard errors are listed in parentheses and clustered by both county and year (two-way clustering). *** *p* < 0.01, ** *p* < 0.05.

## Data Availability

The China Health and Nutrition Survey is available from the University of North Carolina website, https://www.cpc.unc.edu/projects/china (accessed on 25 September 2021).

## Appendix A

**Table A1:** Components of QWB and the corresponding questions and weights in CHNS.

Index	Definition	Variables in CHNS	Weights
MOB Mobility scales			
MOB1	No limitations for health reasons		−0.000
MOB2	Did not drive a car, did not ride in a car as usual, health related	U157-U160, U176	−0.062
MOB3	In hospital, health related		−0.090
PAC Physical Activity Scales			
PAC1	No limitations for health reasons		−0.000
PAC2	Had trouble or did not try to lift, stoop, bend over, or use stairs or inclines, health related	U161-U166	−0.060
PAC3	In bed, chairs, or couch for most or all of the day, health related		−0.077
SAC Social Activity Scales			
SAC1	No limitations for health reasons	U48, U49	−0.000
SAC2	Limited in other role activity, health related	U167, U169	−0.061
SAC3	Limited in major role activity, health related	U171, U173-U175	−0.061
SAC4	Performed no major role activity, health related, but did perform self-care activities	U177	−0.061
SAC5	Performed no major role activity, health related, and did not perform self-care activities	U178	−0.106
CPX	**CPX Symptom/Problem Complexes** Various diseases	U179, U181-U192, U184a, U186a-b, U12-U19, U22, U24a-U24h, U24j, U24n	See following Appendix A Table A2 [58]

Notes: QWB = 1 + CPX + MOB + PAC + SAC. If the individual has multiple diseases and symptoms in the same index, we choose the one with the lowest weight in calculation.

**Table A2:** Components of CPX index.

CPX No.	CPX Description	Weights
1	Death (not on respondent’s card)	−0.727
2	Loss of consciousness such as seizure (fits), fainting, or coma (out cold or knocked out)	−0.407
3	Burn over large areas of face, body, arms, or legs	−0.387
4	Pain, bleeding, itching, or discharge (drainage) from sexual organs- does not include normal menstrual (monthly) bleeding	−0.349
5	Trouble learning, remembering, or thinking clearly	−0.340
6	Any combination of one or more hands, feet, arms or legs either missing, deformed (crooked), paralyzed (unable to move), or broken- includes wearing artificial limbs or braces	−0.333
7	Pain. Stiffness, weakness, numbness, or other discomfort in chest, stomach (including hernia or rupture), side, neck back, hips, or any joints or hands, feet, arms, or legs	−0.299
8	Pain, burning, bleeding, itching, or other difficulty with rectum, bowel movements, or urination (passing water)	−0.292
9	Sick or upset stomach, vomiting or loose bowel movement, with or without fever, chills, or aching all over	−0.290
10	General tiredness, weakness, or weight loss	−0.259
11	Cough, wheezing, or shortness of breath, with or without fever, chills, or aching all over	−0.257
12	Spell of feeling upset, being depressed, or of crying	−0.257
13	Headache, or dizziness, or ringing in ears, or spells of feeling hot, or nervous, or shaky	−0.244
14	Burning or itching rash on large areas of face, body, arms, or legs	−0.240
15	Trouble talking, such as lisp, stuttering, hoarseness, or being unable to speak	−0.237
16	Pain or discomfort in one or both eyes (such as burning or itching) or any trouble seeing after correction	−0.320
17	Overweight for age and height or skin defect of face, body, arms. Or legs, such as scars, pimples, warts, bruises, or changes in color	−0.188
18	Pain in ear, tooth, jaw, throat, lips, tongue; several missing or crooked permanent teeth- includes wearing bridges or false teeth; stuffy, runny nose; or any trouble hearing- includes wearing a hearing aid	−0.170
19	Taking medication or staying on a prescribed diet for health reasons	−0.144
20	Wore eyeglasses or contact lenses	−0.101
21	Breathing smog or unpleasant air	−0.101
22	No symptoms or problem (not on respondent’s card)	−0.000

**Table A3:** Second-stage estimation: Effect of PM_2.5_ concentrations on productivity using only the common set of observations (*n* = 9175).

	Annual Working Hours		Working Months		Average Hourly Wage	
	(1) OLS	(2) 2SLS	(3) OLS	(4) 2SLS	(5) OLS	(6) 2SLS
						
PM_2.5_ (μg/m^3^)	0.711 (2.577)	−27.88 *** (12.63)	0.005 (0.010)	−0.047 * (0.025)	−0.009 (0.053)	−0.34 ** (0.17)
KP F-statistic		35.64		36.14		35.08
Mean of Dep	1678	1678	9.588	9.588	6.98	6.98
S.D. of Dep	904.8	904.8	3.134	3.134	13.26	13.26
Individual FE	Yes	Yes	Yes	Yes	Yes	Yes
Year FE	Yes	Yes	Yes	Yes	Yes	Yes
Weather controls	Yes	Yes	Yes	Yes	Yes	Yes
Individual controls	Yes	Yes	Yes	Yes	Yes	Yes
Observations	9376	9376	9376	9376	9376	9376

Notes: The dependent variables are annual working hours in the last year in columns (1) and (2), working months in the last year in columns (3) and (4), and average hourly wage in the last year in columns (5) and (6). We exclude individuals who earn the top 0.3% and the bottom 0.3% in columns (5) and (6) (hourly wage) to avoid outlier bias. Panel A reports 2SLS estimates, in which we use the number of thermal inversions as an instrument for PM2.5. Panel B reports the OLS estimates which air pollution is not instrumented. Weather controls include 5 °C temperature bins, second-order polynomials in average snow thickness, vapor pressure, wind speed, sunshine duration, relative humidity, cumulative precipitation rain duration, and precipitation solid duration for the last year. Individual controls include variables indicating the individual’s marital status, retired or not, etc. Standard errors are listed in parentheses and clustered by both county and year (two-way clustering). *** *p* < 0.01, ** *p* < 0.05, * *p* < 0.1.

**Table A4:** Selected Control Variables’ Performance in Table 3.

		Productivity Measures	
	(1) Annual Working Hours	(2) Working Months	(3) Average Hourly Wage
PM_2.5_(μg/m^3^)	−26.60 *** (10.09)	−0.082 ** (0.033)	0.247 *** (0.057)
Retirement (=1 retired; =0 in-service)	−148.49 ** (63.99)	−1.311 *** (0.245)	−0.933 (0.930)
Temperature Bins (Days)			
(35 °C, 40°]	−18.22 ***	−0.023 *	−1.358
	(6.52)	(0.012)	(0.950)
(30 °C, 35°]	−12.51 ***	−0.021 ***	−1.040 *
	(2.80)	(0.005)	(0.602)
(−25 °C, −30°]	−27.87 ***	−0.400 ***	−0.992 *
	(13.97)	(0.080)	(0.602)
(−30 °C, −35°]	−8.352 ***	0.186	−0.948
	(3.421)	(0.46)	(0.605)
Individual FE	Yes	Yes	Yes
Year FE	Yes	Yes	Yes
Observations	18,346	18,711	9376

Notes: The dependent variable is annual local county PM2.5 concentrations in the last year. Thermal inversions are aggregated from every 6 h to 12 months for the last year. To avoid outlier bias, we exclude individuals who earn the top 0.3% and the bottom 0.3% in column (3) (hourly wage). Weather controls include 5 °C temperature bins, second-order polynomials in average snow thickness, vapor pressure, wind speed, sunshine duration, relative humidity, cumulative precipitation rain duration, and precipitation solid duration for the last year. Individual controls include variables indicating the individual’s marital status, retired or not, etc. Standard errors are listed in parentheses and clustered by both county and year (two-way clustering). *** *p* < 0.01, ** *p* < 0.05, * *p* < 0.1. The numbers of observations in columns (1) and (3) are different because of missing dependent variables, especially hourly wage. The results match our expectations well and suggest that retirement status and more extremely hot and cold days negatively impact both working time and income.

**Table A5:** Effect of Air Pollution on Productivity by Different Education Level.

	Sub-Group Models by Highest Education Level Achieved				
	(1) Primary School	(2) Secondary School	(3) High School	(4) Technical or Vocational School	(5) College Degree or Higher
**Annual working hours**					
PM_2.5_ (μg/m^3^)	−11.03 * (7.166)	−24.72 * (14.62)	−47.25 ** (20.53)	−33.59 * (20.06)	−43.26 (25.20)
Mean of Dep	1363.49	1725.81	1940.99	1996.62	1750.68
S.D. of Dep	1027.51	1042.35	874.01	662.29	810.96
KP F-statistic	16.39	28.35	25.39	36.30	62.92
Observations	3762	8553	5376	1957	1615
**Working months**					
PM_2.5_ (μg/m^3^)	−0.034 (0.074)	−0.083 ** (0.041)	−0.102 ** (0.052)	−0.083 ** (0.041)	−0.033 (0.048)
Mean of Dep	8.685	9.599	10.613	11.37	10.53
S.D. of Dep	3.485	3.310	2.779	1.906	2.853
KP F-statistic	15.45	30.01	26.87	17.39	43.381
Observations	4409	8781	3680	1981	1623
**Hourly wage**					
PM_2.5_ (μg/m^3^)	−0.017 (0.019)	−0.014 (0.012)	−0.010 (0.015)	−0.020 * (0.011)	−1.03 *** (0.42)
Mean of Dep	5.23	6.25	6.52	6.67	9.45
S.D. of Dep	10.63	14.43	13.06	6.86	15.43
KP F-statistic	15.92	28.76	29.70	12.41	24.60
Observations	1227	3135	2475	1769	2308

Notes: The dependent variables are annual working hours in the last year in Section 1, working months in the last year in Section 2, and average hourly wage in the last year in Section 3. To avoid outlier bias, we exclude individuals who earn the top 0.3% and the bottom 0.3% in Section 3 (hourly wage). Regression models are estimated separately for each subsample. All the regressions report 2SLS estimates with controls. Weather controls include 5 °C temperature bins, second-order polynomials in average snow thickness, vapor pressure, wind speed, sunshine duration, relative humidity, cumulative precipitation rain duration, and precipitation solid duration for the last year. Individual controls include variables indicating the individual’s marital status, retired or not, etc. Standard errors are listed in parentheses and clustered by both county and year (two-way clustering). *** *p* < 0.01, ** *p* < 0.05, * *p* < 0.1.

**Table A6:** Estimates on Annual Salary.

	Baseline		Education		Skills		Workplace	
	(1) OLS	(2) 2SLS	(3) <College	(4) ≥College	(5) High Skilled	(6) Low Skilled	(9) Indoor	(10) Outdoor
PM_2.5_ (μg/m^3^)	−31.65 (60.16)	−485.54 ** (240.35)	−431.38 * (220.84)	−1378.66 ** (661.160)	−192.04 (374.73)	−505.98 * (261.91)	−363.58 (242.02)	−682.76 (447.07)
Mean of Dep	17,175.32	17,175.32	15,416.25	23,004.11	21,100.07	16,335.5	15,672.43	17,614.84
S.D. of Dep	18,795.81	18,795.81	17,083.60	22,644.49	20,375.97	18,332.35	18,043.02	18,988.77
KP F-statistic		41.40	36.89	24.40	26.72	35.45	38.86	30.26
Observations	9348	9348	7064	2284	1733	7615	6990	2358

Notes: The dependent variable is the last year’s annual salary, which equals the product of annual working hours times the hourly wage. To avoid outlier bias, we exclude individuals who earn the top 0.3% and the bottom 0.3% in salary. Regression models are estimated separately for each subsample. Columns (2)–(9) report 2SLS estimates with controls. Weather controls include 5 °C temperature bins, second-order polynomials in average snow thickness, vapor pressure, wind speed, sunshine duration, relative humidity, cumulative precipitation rain duration, and precipitation solid duration for the last year. Individual controls include variables indicating the individual’s marital status, retired or not, etc. Standard errors are listed in parentheses and clustered by both county and year (two-way clustering). ** *p* < 0.05, * *p* < 0.1.
